# Refreshing boosts memory but does not protect it against interference

**DOI:** 10.3758/s13423-026-02856-0

**Published:** 2026-02-17

**Authors:** Caro Hautekiet, Evie Vergauwe, Alessandra S. Souza

**Affiliations:** 1 Faculty of Psychology and Educational Sciences, University of Geneva, Geneva, Switzerland; 2 Department of Psychology, University of Zurich, Zürich, Switzerland; 3 Center for Psychology, Faculty of Psychology and Education Sciences, University of Porto, Porto, Portugal

**Keywords:** Working memory, Attention, Focus of attention, Refreshing, Interference

## Abstract

Attention can be directed to representations maintained in working memory, increasing their accessibility for later retrieval. Previous studies have observed a cumulative benefit of focused attention: The more often an item is brought into the focus of attention, the better it is recalled. This can be explained by the notion that a memory representation is strengthened every time attention is focused on it, a process known as refreshing. The current study investigated whether refreshing goes together with increased robustness against interference. In two experiments, we examined whether an item’s susceptibility to interference by an interruption task gradually decreases as an item is more frequently refreshed. Participants memorized six colors for a continuous reproduction test. During the retention interval, they were instructed to refresh some of these colors once, twice, or never, and then completed an interruption task in half of the trials. In Experiment 1, the interruption was a domain-non-overlapping auditory parity judgment task, whereas in Experiment 2, we used a domain-overlapping visual color judgment task. In both experiments, memory performance was hindered by the interruption but improved with increasing refreshing frequency. These two effects did not interact, indicating that the negative impact of the interruption was not reduced by refreshing. Thus, although refreshing an item improves retrieval from working memory, it does not provide greater protection against interference.

**Supplementary Information:**

The online version contains supplementary material available at https://doi.org/10.3758/s13423-026-02856-0.

In many day-to-day situations, we need to keep bits of information temporarily in mind to complete a task. To do so, we often rely on a limited-capacity cognitive system that is in charge of processing and keeping information accessible over a short period of time (i.e., working memory; see Oberauer, 2019, for a recent review). Working memory can only hold a handful of items accessible, but each item can be selectively processed and manipulated by directing attention to it (e.g., Garavan, 1998; Oberauer, 2002). Past studies consistently demonstrated that memory performance is improved for focused information compared with other information in working memory (e.g., Barrouillet et al., 2007; Griffin & Nobre, 2003; Hautekiet et al., 2025a, 2025b; Souza et al., 2016). This may be explained by the *strengthening hypothesis*, which proposes that attending to information in working memory strengthens the binding between an item and its context, thereby facilitating recall (e.g., Kuo et al., 2011; Rerko & Oberauer, 2013). Indeed, a robust finding in the literature demonstrates that information is remembered more precisely the more frequently it receives attention during retention (e.g., Souza et al., 2015, 2018; Souza & Oberauer, 2017; Vergauwe et al., 2024). In these studies, attention is directed towards specific information in working memory by means of *refreshing*, which consists of the act of ‘thinking back to’ a specific representation in working memory.

Refreshing is often studied using the instructed refreshing paradigm (e.g., Loaiza & Souza, 2022; Souza et al., 2015, 2018; Souza & Oberauer, 2017; Vergauwe et al., 2024). For example, Souza et al. (2015) presented participants with six to-be-memorized colored disks, of which one had to be reproduced at the end of the trial using a color wheel. During retention, participants were presented with four sequential refreshing cues, each pointing to a memory location. Participants were instructed to think back to the memory item presented in the cued location (i.e., by attending to it without verbally rehearsing it). At test, the probe could be an item that was not refreshed, that was refreshed once, or that was refreshed twice. Souza and colleagues observed a decrease in recall error as an item had been refreshed more often. This improvement in memory performance can be explained by the idea that representations—and their contexts—are strengthened in memory each time attention is directed towards them (e.g., Kuo et al., 2011; Rerko & Oberauer, 2013; see Souza & Oberauer, 2016, for a review). If representations are strengthened by refreshing, a reasonable question is whether these representations also become more robust against interference. To our knowledge, whether refreshing has a protective effect against interference has not been directly investigated in prior studies. We have, however, evidence from studies in which participants could focus their attention continuously on a single working memory content, the so-called *retro-cue effect*.

In retro-cue studies, a single cue is presented during the retention interval, indicating this item as relevant to the memory test. Tests of the retro-cued item show improved performance: responses are faster and more accurate than in baseline conditions in which attention is distributed among all items (see Souza & Oberauer, 2016, for a review). Critically, the retro-cued item is often (but not always) observed to be protected against interference (e.g., Makovski & Jiang, 2007; Makovski & Pertzov, 2015; Souza et al., 2016; Vergauwe et al., 2025). Across these studies, a distinction can be made between two types of interference: participants can be presented with either (1) a to-be-ignored, irrelevant stimulus, which we will refer to as *passive distraction*, or (2) a stimulus requiring active engagement and attention, which we will refer to as *active interruption* (see Clapp & Gazzaley, 2011; Clapp et al., 2010; Janowich et al., 2015; Marsh et al., 2024; Vergauwe et al., 2009; Zickerick et al., 2020 for a similar distinction). For passive distraction, mixed findings have been observed. While some studies found that retro-cued items are protected from this type of interference (e.g., Schneider et al., 2017; van Moorselaar et al., 2015; Vergauwe et al., 2025), others observed equal vulnerability for retro-cued and not-cued items (e.g., Hautekiet et al., 2025a, 2025b; Jeanneret et al., 2024, Experiments 2–3). Several recent studies even observed that working memory representations were not impacted by this passive form of interference at all, regardless of being retro-cued or not (e.g., Hautekiet et al., 2025a, 2025b; Jeanneret et al., 2024, Experiment 1; Zhang & Lewis-Peacock, 2023b). Because working memory does not appear to be consistently affected by passive distraction, this type of interference is not ideally suited to investigate whether refreshing results in stronger, more robust representations.

In contrast, the findings for active interruption have proven to be more consistent. Different studies observed that retro-cued items are protected from interruption, whereas other information in working memory remains vulnerable (e.g., Loaiza & Souza, 2019; Makovski & Pertzov, 2015; Rerko et al., 2014; Souza et al., 2016). For example, Makovski and Pertzov (2015) presented participants with three to-be-memorized orientations of which one had to be reproduced at the end of the trial. The encoding display was followed by a retro-cue, which validly indicated the to-be-tested orientation. In between the cue and the test display, an interruption could be presented. On interruption trials, participants were auditorily or visually presented with a digit and asked to judge whether it was odd or even by pressing one of two keys. The interruption hindered memory performance in the no-cue trials, but this negative effect was largely reduced for the retro-cued item regardless of whether the digit was presented auditorily or visually.

The retro-cue effect can be explained by several mechanisms, with refreshing being one of several contenders (see Souza & Oberauer, 2016, for a full overview). The instructed refreshing paradigm (Souza et al., 2015), on the other hand, was specifically developed to isolate the contributions of strengthening due to focused attention. There are several outstanding differences between the retro-cue and the refreshing paradigm. In retro-cue studies, the cued item can be maintained continuously in the focus of attention, it can be strengthened, its retrieval can be started ahead of testing, and non-cued items can be removed from working memory, which releases capacity.^1^ Conversely, in the refreshing paradigm, the focus of attention has to cycle over several memory representations, with all representations maintaining their potential relevance for the test. This means that no single item can be retained in the focus of attention, and representations cannot be removed or retrieved in advance of the test situation, leaving only strengthening as a mechanism for improved performance. As such, the instructed refreshing task serves as the ideal paradigm to assess the hypothesis that refreshing protects against interference. The goal of the present study is to assess this possibility.

## The current study

**Fig. 1:**
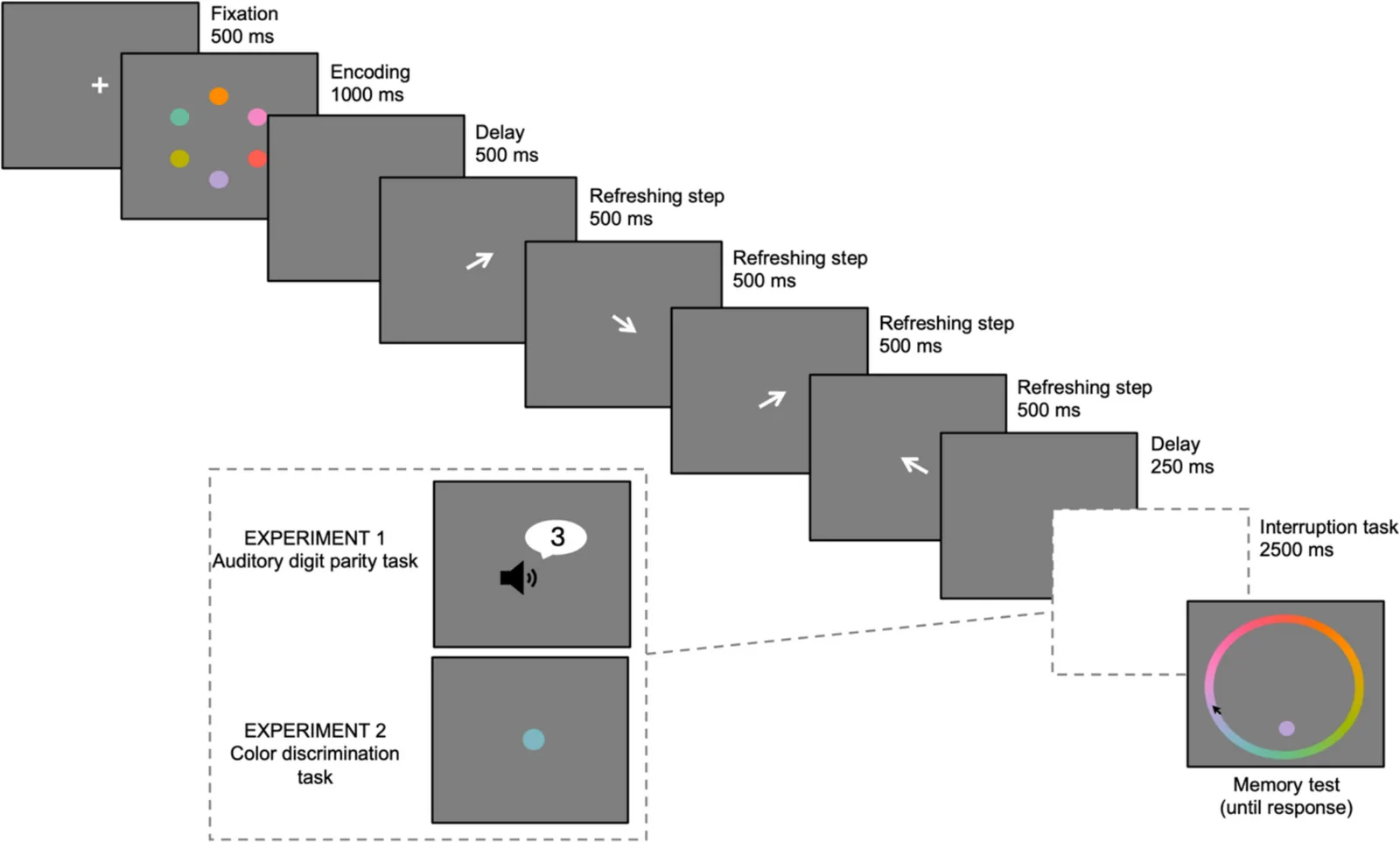
Illustration of the flow of events in Experiments 1 and 2

To investigate whether refreshing protects against interference, we used a paradigm similar to the one developed by Souza et al. (2015, Experiment 1). We focus on active interruptions as our interference manipulation, given that passive distraction has produced inconsistent results. Our general task setup was as follows. Participants memorized six colored disks, one of which had to be reproduced at the end of the trial using a color wheel (see Fig. 1). In between, a sequence of four refreshing cues instructed participants to “think back” to the memory item presented in the cued location (following the same procedure as Souza et al., 2015). The last refreshing cue was followed by an interruption task in 50% of the trials. In Experiment 1, the interruption consisted of an auditory digit to be classified as odd or even (i.e., a verbal task). This task was chosen to specifically load central attention (see, e.g., Barrouillet et al., 2007; Makovski & Pertzov, 2015; Vergauwe et al., 2014)—while minimizing perceptual or domain-overlapping interference—allowing us to isolate central attentional effects.^2^ Additionally, previous studies showed that it impairs visual working memory for colors (e.g., Loaiza & Souza, 2019; Souza & Oberauer, 2017), which is our main task. To foreshadow our results, we replicated the refreshing frequency benefit, with better memory for items that were refreshed more often, and found memory to be disrupted by the interruption task. However, this disruptive effect did not differ between items that were refreshed once, twice, or never.

Experiment 1 showed that refreshing does not protect the attended information from domain-non-overlapping interference. Still, it is possible that a stronger interruption task, one that includes domain-overlapping interference over and above competition for central attention, might reveal protection at a representation-specific level (e.g., from distortions in color space). Therefore, in Experiment 2, we replaced the interruption task with a visual color judgment task (based on Rerko et al., 2014). This task includes a central attention demand as well as domain-specific interference. Replicating Experiment 1, we observed main effects of refreshing and interference, but no interaction. Hence, refreshing did not convey protection from domain-specific interference either.

The experimental files, raw data, and analysis scripts can be found on the Open Science Framework (OSF; https://osf.io/94rvx/overview). The protocol of both experiments (sample size justification, hypotheses, analyses and data exclusions) was preregistered prior to conducting the studies, on the OSF (Experiment 1: https://osf.io/nuvyh/overview and Experiment 2: https://osf.io/7hd8e/overview). Experiment 2 was conducted after Experiment 1, but given their similarities, the methods and results are reported together.

## Method

### Participants and design

For both experiments, participants were undergraduate students from the University of Geneva and the University of Porto who participated in exchange for course credits. All participants completed an online informed consent form before participating, and approval was obtained from the ethical commission at the University of Geneva and the University of Porto. In both experiments, the design consisted of a 3 × 2 factorial design, with refreshing frequency (three levels; 0-refreshed, 1-refreshed, and 2-refreshed) and interruption task (two levels; present vs. absent) as within-subjects variables.

The number of participants was determined using Bayesian sequential hypothesis testing in both experiments. Specifically, we preregistered to stop data collection after a first batch of a minimum of 60 participants^3^ (at least 30 from each site) if a BF ≥ 10 was reached for or against the critical interaction between refreshing frequency and interruption task. If this would not be the case, data of a second batch of at least 60 participants (at least 30 from each site) was planned to be collected (resulting in a total of at least 120 participants).

In total, 86 participants^4^ took part in Experiment 1; 42 participants from the University of Geneva (36 women, five men, one other, mean age = 23 years) and 44 participants from the University of Porto.^5^ As preregistered, we excluded participants with less than 55% accuracy on the parity judgment task (i.e., to exclude participants that were randomly guessing).^6^ This resulted in a final sample of 78 participants; 36 participants from the University of Geneva and 42 participants from the University of Porto.

In Experiment 2, a total of 70 participants took part; 35 participants from the University of Geneva (32 women, three men, mean age = 21 years) and 35 participants from the University of Porto. As preregistered, we excluded participants with less than 55% accuracy on the color judgment task. This resulted in a final sample of 62 participants: 34 participants from the University of Geneva and 28 participants from the University of Porto. All participants were tested online.

### Materials

In both experiments, the to-be-memorized colors were randomly sampled from a color wheel defined in the CIELAB color model (with a = 20, b = 38, L = 70, and radius = 60), with the constraint that the colors had to differ from each other by a minimum distance of 20°. The color wheel was randomly rotated from trial to trial to avoid any color–position association learning.

For the digit parity task used in Experiment 1, all digits between 1 and 8 were used. We downloaded the audio files for the digits from https://ttsmp3.com/ pronounced in French (voice ‘Céline’) and pronounced in Portuguese (voice ‘Inês’). Each file was adapted, using Audacity (https://www.audacityteam.org/), to ensure that all files had a similar duration of about 400 ms.

For the interruption task used in Experiment 2, we preselected eight colors from the color wheel in such a way that, like in Experiment 1, we had four items for each response category (four ‘warm’ colors and four ‘cold’ colors). The selection of the colors per category was based on prior studies that used the same warm/cold distinction (e.g., Koo & Kwak, 2015; Ou et al., 2004; Rerko et al., 2014; Souza et al., 2021). The interruption color was selected from the same pool as the memory items, but it was not included in the memory set on the same trial (i.e., ‘plausible’ interference item; see, e.g., Hu et al., 2014; Ueno et al., 2011). Specifically, the color selected for the interruption task differed at least 20° from any of the memory items (i.e., the same distance as between the different memory items). The selected colors, as well as their corresponding RGB codes, can be found on OSF (https://osf.io/d7j4q).

### Procedure

Both experiments were programmed using *lab.js* (Version 23.0-alpha2; Henninger et al., 2022) and data collection was done online using a JATOS server. Participants accessed the study via a link and were asked to execute the experiment on a computer and not a tablet or phone.

For both experiments, we followed a similar procedure as in Experiment 1 by Souza et al. (2015). Each trial started with a central white fixation cross presented against a grey background for 500 ms. Next, six colored disks were presented simultaneously for 1,000 ms (see Fig. 1). At the end of the trial, the test display was presented. The test display consisted of a color wheel (randomly rotated from trial to trial) together with a probe disk indicating the target location. The color of the probe disk changed as participants moved the mouse around the wheel. At the first presentation of the color wheel, the probe disk immediately took on the color of the mouse position. Participants were instructed to reproduce the corresponding color of the probe disk by clicking on a point in the color wheel. The test display remained on screen until a response had been provided or until 20 s had elapsed. Thereafter, an intertrial interval of 500 ms followed. Participants were instructed to respond as accurately as possible (speed was not emphasized). Trials were self-paced and started as soon as participants pressed the ‘space’ button. Specifically, participants were instructed to put their left index finger on the ‘d’ key, put their right index finger on the ‘k’ key^7^, and press ‘space’ using their thumb to start the next trial.

After the presentation of the memory set, the refreshing manipulation was inserted. Specifically, four centrally located arrows were sequentially presented for 500 ms each. The first arrow was presented 500 ms after the offset of the memory set. As in Souza et al. (2015), we will refer to the presentation of each cue as a refreshing step. For each refreshing step, participants were asked to think back to the item previously presented in the indicated location. Participants were made aware of the fact that these arrows do not reliably indicate which item will be tested, but they were still strongly encouraged to think of the indicated item. As in Souza et al. (2015), there were five possible sequences of the refreshing steps. If A, B, C, and D represent a random subset of the memory items: (1) the four arrows could point to four different items (A-B-C-D), (2) two arrows could point to two different items, and two arrows point to the same item (A-B-A-C, A-B-C-B; A-B-C-A), and (3) two arrows could point to one item and the two other arrows point to another item (A-B-A-B). It is important to note that across two successive refreshing steps, the arrows always pointed to different items. Thus, the same item could not be cued twice in a row.

Following these constraints, the following refreshing frequencies were possible: items that were not cued and thus not refreshed (0-refreshed items), items that were cued once (1-refreshed item), and items that were cued twice (2-refreshed items). When an item was cued twice, the item was cued once in Cue Position 1 or 2 and once in Cue Position 3 or 4. Items that were cued only once could be cued in any of the cue positions. Thus, when an item was cued twice, the items either had a shorter interval between memory-display offset and the first cue or between the last cue and the test-display onset.^8^ The sequence of the cues was chosen randomly on each trial. The target of recall was always selected from the 0-, 1-, or 2-refreshed items, and each refreshing condition was tested an equal number of times.

In half of the trials, participants were presented with an interruption task between the last refreshing step and the test display. In Experiment 1, participants were presented with an auditory parity judgment task. Specifically, one digit was auditorily presented and participants were asked to indicate whether the digit was odd (‘d’ key) or even (‘k’ key) as fast as possible (within max 2,500 ms). If participants responded before the response time ended, the screen remained blank until a total of 2,500 ms had passed (i.e., response time + blank). In the other half of the trials (Interruption absent trials), the 2,500 ms interval remained blank. Thus, the time between the last refreshing step and the test display remained the same regardless of whether the interfering task was presented or not.

In Experiment 2, participants were presented with a visual color judgment in a random half of the trials (as done in Rerko et al., 2014). That is, one colored circle was centrally presented on screen for 1,300 ms. During this time, participants were instructed to judge whether the color was warm (‘d’ key) or cold (‘k’ key). The colored circle remained on screen for the total duration of 1300 ms, even when a response had been provided before that time. Following the interruption task offset, the screen remained empty for 1,200 ms (to keep the same total retention interval time as in Experiment 1). On interruption-absent trials, the screen remained empty for a total of 2,500 ms.

In both experiments, participants completed 210 trials divided into five blocks of 42 trials. Of the 210 trials, half included an interruption task and half did not (105 each). Within these trials, there was an equal division of each refreshing condition (35 trials each). The different trial combinations were presented randomly intermixed with the constraint that each block consisted of 21 trials with and 21 trials without the interruption task (each including seven trials per refreshing condition). In between each block, participants could take a small break. Before starting the experimental trials, participants received instructions on the different parts of the task, with practice trials on each part of the task as well as combined (see Supplementary Materials for more details).

Given that participants completed the study on their own devices, we included a sound (Experiment 1) and brightness test (Experiment 1 & 2) before the start of the experiment. Participants were instructed to adjust the sound and brightness of the screen to a comfortable level and to maintain it on this level for the remainder of the study. Additionally, during each break in Experiment 1, participants were reminded to make sure their sound was activated before continuing the experiment. After these settings, the study was set to full-screen mode. Contrary to the original study by Souza et al. (2015), the current study did not include articulatory suppression.^9^

## Analysis and results

All analyses were preregistered prior to data collection and executed using the *BayesFactor* package (Morey & Rouder, 2018) in R with default settings. Our measure of interest was the absolute distance in degrees between the target color and the response color on the color wheel, referred to as recall error. The results and discussion of the preregistered exploratory analyses are reported in the Supplementary Materials (https://osf.io/d7j4q) .

**Fig. 2:**
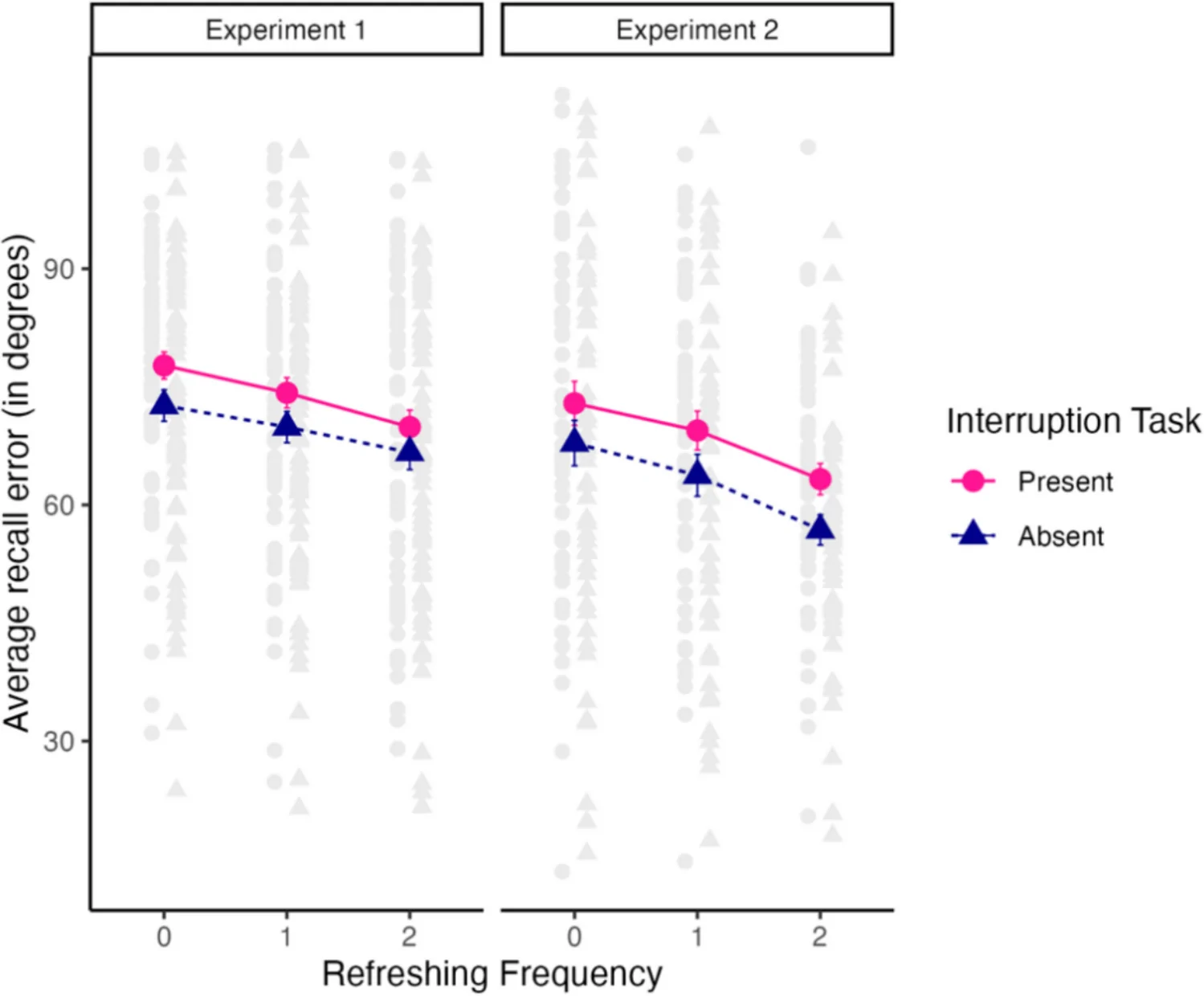
Average recall error (in degrees) for Experiments 1 and 2, as a function of refreshing frequency and interruption task (domain-non-overlapping in Experiment 1, domain-overlapping in Experiment 2), presented with standard error bars and individual scores (grey). (Color figure online)

In both experiments, we ran a Bayesian repeated-measures analysis of variance (ANOVA) on averaged recall error, with refreshing frequency (three levels; 0-refreshed, 1-refreshed, 2-refreshed) and interruption task (two levels; present, absent) as within-subjects variables. The results are reported in Table 1. For both experiments, the best model included the two main effects and including the interaction resulted in substantially worse models (Bayes factors of about 17 against the interaction of interest). Thus, in both experiments, memory performance improved the more often an item had been refreshed, and it was hindered by the presence of the interruption task, but these two effects did not interact (see Fig. 2).

**Table 1.**
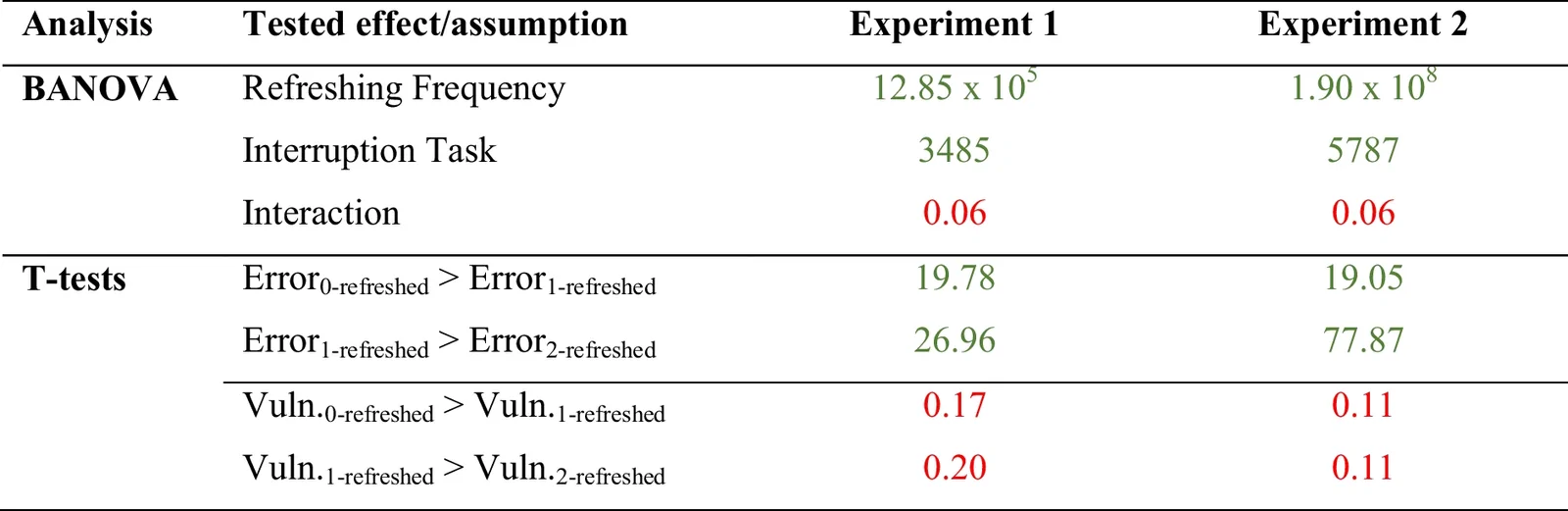
Bayes factors (BF_10_) for the preregistered Bayesian ANOVA (BANOVA) and *t* tests on averaged recall error and vulnerability scores

Bayes factors (BFs) resulting from model comparison between the best model and the model excluding the effect/interaction (if it is included in the best model) or including the effect/interaction (if it is not part of the best model). For the BANOVA, the BFs reflect evidence in favor of or against a main effect or interaction, while for the *t* tests, the BFs reflect evidence in favor of or against the tested assumption. The ‘Interaction’ row refers to the comparison of the full model to the best model (which was the main effects-only model in both experiments). When the BF presents conclusive evidence in favor of the tested effect (BF_10_ > 3), the text is presented in green; when the BF reflects conclusive evidence against the tested effect (BF_10_ < 0.33, which corresponds to BF_01_ > 3), the text is presented in red. Vuln. = Vulnerability Score.

**Fig. 3:**
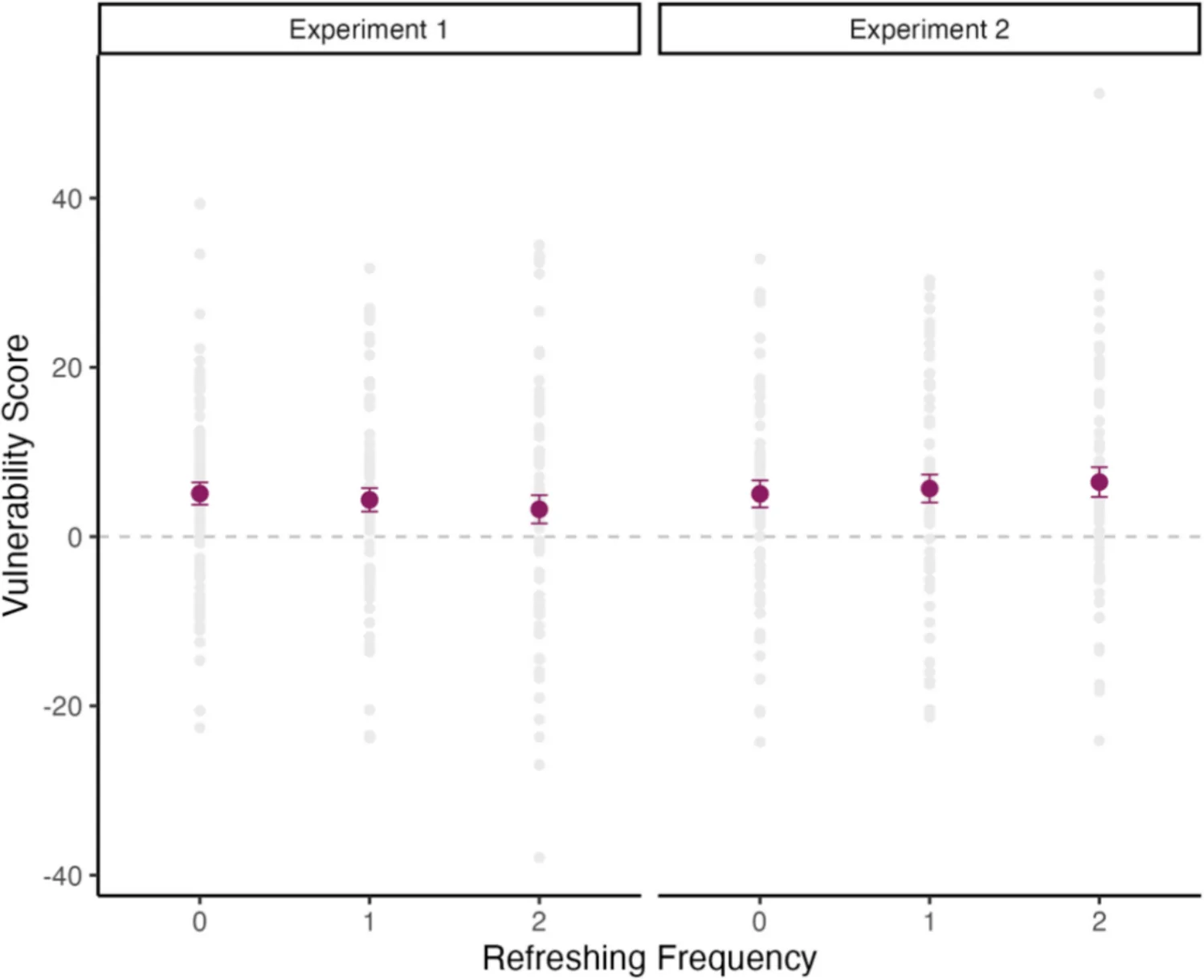
Vulnerability scores (recall error interruption present – recall error interruption absent) for Experiments 1 and 2, as a function of refreshing frequency, presented with standard error bars and individual scores (grey). A vulnerability score above 0 (grey dotted line) means that memory performance got worse in the presence of an interruption task, whereas a vulnerability score below 0 means that memory performance got better in the presence of an interruption. If memory performance would be unaffected by the interruption task, the vulnerability score should be 0

Furthermore, we ran two sets of two Bayesian paired-samples one-sided *t* tests. The first set tested whether recall error is larger for the 0-refreshed items compared with the 1-refreshed items, and for the 1-refreshed items compared with the 2-refreshed items. The second set measured whether the vulnerability score (recall error interruption present minus recall error interruption absent) is larger for 0-refreshed items compared with 1-refreshed items, and for 1-refreshed items compared with 2-refreshed items. The results are presented in Table 1. The results of the first set of *t* tests are consistent with the notion that recall error decreases as the representation has been refreshed more often. The results of the second set of t-tests indicate that the memory representation does not become more robust against interference the more often it has been refreshed (see Fig. 3), regardless of whether we used a domain-non-overlapping interruption task (Experiment 1) or a domain-overlapping interruption task (Experiment 2).

## Discussion

The current study investigated whether refreshing information more frequently strengthens memory representations and reduces susceptibility to interference. Memory performance increased with refreshing frequency, replicating prior results (e.g., Loaiza & Souza, 2022; Souza et al., 2015, 2018; Souza & Oberauer, 2017; Vergauwe et al., 2024). Additionally, an interruption task incurring either domain-non-overlapping (Experiment 1) or domain-overlapping processing (Experiment 2) impaired working memory performance. However, and most importantly, the negative impact of the interruption task was not modulated by how often an item had been refreshed. Thus, more frequently refreshed information becomes strengthened but does not become less susceptible to an interruption task.

Furthermore, our preregistered exploratory analyses, available in the Online Supplementary Materials, demonstrated that this conclusion holds regardless of the refreshing position of the tested item. With this analysis, we tested whether the last refreshed item—which presumably resided in the focus of attention at the moment of the interruption—is less susceptible to interference, similar to reports from retro-cue studies (e.g., Makovski & Jiang, 2007; Souza et al., 2016; van Moorselaar et al., 2015). Our findings clearly demonstrated that the impact of the interruption task was not modulated by when the item had been refreshed. Again, this was the case regardless of whether we used a central, domain-non-overlapping interruption task (Experiment 1) or a perceptual, domain-overlapping interruption task (Experiment 2).

A relatively recent meta-analysis (Morey, 2018) showed that dual-task costs in visual working memory are of similar magnitude for domain-non-overlapping and domain-overlapping interference, suggesting that interruption tasks from a different domain (e.g., verbal) and from the same domain (e.g., visual) would be similarly effective to disrupt memory. In line with this conclusion, we did not observe greater disruption when the interfering task was domain-overlapping compared to when it was not. An exploratory Bayesian *t* test on the difference scores (performance on interruption present trials minus performance on interruption absent trials) resulted in moderate evidence against a difference between Experiment 1 and 2 (BF_10_ = 0.21).

One could argue that the absence of a protective state for the focus of attention in our study is due to the specific mixed-trial design, as many prior studies used a blocked design (e.g., Loaiza & Souza, 2019; Makovski & Pertzov, 2015). However, as the protective state can be both present (e.g., Rerko et al., 2014) or absent (current findings) with a mixed-trial design, and can be both present (e.g., Souza et al., 2016) or absent (e.g., Jeanneret et al., 2024) with a blocked design, the design is unlikely to explain the absence of a protective state. Alternatively, one could argue that we did not observe a protective effect because participants had sufficient time to recover from the interruption and refocus attention before memory was tested, as we included a fixed duration of 2,500 ms for the interruption task. However, we find this explanation insufficient because the interruption task credibly hindered performance similarly across all refreshing levels. Hence, even if participants could repair their working memory representations postinterruption, this would involve a postinterference activity unrelated to our refreshing manipulation.

Thus, overall, our findings indicate that (a) refreshing increases the accessibility of focused information, (b) the differential accessibility conveyed by refreshing is not lost after an interruption task that engages the focus of attention, and (c) the interruption task has a detrimental effect on memory performance overall. The latter probably occurs because the interruption task consumes central attention, as we did not observe any additional contribution of domain-specific interference to forgetting. This effect is in line with the so-called cognitive load effect (e.g., Barrouillet et al., 2007) and with propositions that this effect reflects the costs of engaging the focus of attention with task processing, which prevents maintenance processes, such as refreshing, from being carried out. This is in line with the proposal of the time-based resource-sharing model (e.g., Barrouillet & Camos, 2014). During the period in which the focus is occupied, information loses accessibility. As all items lose accessibility similarly over time, no interaction between refreshing and the interruption effect is observed.

Our findings are in contrast with the ones observed for retro-cued items, which are generally shielded against the negative effects of active interruptions (e.g., Makovski & Pertzov, 2015; Rerko et al., 2014; Souza et al., 2016; Zhang & Lewis-Peacock, 2023a). As we outlined in the introduction, retro-cue effects can be explained by different mechanisms other than refreshing (see Souza & Oberauer, 2016). One mechanism could be *persistent maintenance in the focus of attention.* The focus of attention might convey a protective state compared to the state of other items maintained in the central region of working memory. Our supplementary analysis renders this possibility unlikely because the last refreshed item—which was arguably in the focus of attention—was not protected from interference. Another mechanism is *retrieval headstart*—the cued item is retrieved in advance of the test situation (and hence ahead of the interference). This could reduce the possibility that interference would get confused with the memory item or that it would damage the memory representation. Finally, a third mechanism is *removal of noncued items from working memory*. This would release working memory capacity that could be used to reduce the competition between memory maintenance and processing of the interruption task. Our findings indicate that one of these mechanisms might explain why retro-cued information is insulated from interference by interruption, whereas strengthening through refreshing does not contribute to interference protection.

Our findings are also in contrast with the pattern we initially expected based on the literature. Still, this is not the first time that the protective effect of the focus of attention was not observed. Specifically, several recent studies found that focused attention improves performance but does not drastically impact an item’s susceptibility to interference (Hautekiet et al., 2025a, 2025b; Jeanneret et al., 2024; Vergauwe et al., 2025; Zhang & Lewis-Peacock, 2023b). Taken together, these studies and the present study suggest that bringing information into the focus of attention does not make it less susceptible to interference, whether this is achieved by means of (1) being the last-presented item (Hautekiet et al., 2025a, 2025b; Vergauwe et al., 2025), (2) being retro-cued (Hautekiet et al., 2025a, 2025b; Zhang & Lewis-Peacock, 2023b), (3) being highly rewarded (Jeanneret et al., 2024; Vergauwe et al., 2025; Zhang & Lewis-Peacock, 2023b), or (4) being refreshed on one or two occasions (Experiments 1 and 2). If the focus of attention does not consistently protect information from interference, we need to reevaluate the presumed generality of its protective function.

In conclusion, our findings demonstrate that focusing attention on specific information in working memory can strengthen the memory representation and thereby improve its retrieval from working memory, but this strengthening does not result in protection from interference. This suggests that the attention-based memory boost and the protective effect, when present, may be independent of each other and rely on distinct mechanisms.

## Supplementary Information

Below is the link to the electronic supplementary material.

Supplementary file1 (DOCX 298 KB)

## Data Availability

The raw data, experimental files, and analysis code are available on the Open Science Framework (OSF): https://osf.io/94rvx/overview Both experiments were preregistered on OSF: Experiment 1: https://osf.io/nuvyh/overview Experiment 2: https://osf.io/7hd8e/overview

## Footnotes

1 The term *removal* is used broadly here, covering both passive decay and active removal accounts (see Lewis-Peacock et al., 2018, for a review).

2 A relatively recent review on attentional refreshing in working memory suggested that “*refreshing depends on central attention, and may operate in a parallel and noninterfering manner with perceptual attention tasks that do not draw upon central resources*” (Camos et al., 2018, p. 7).

3 Because of course regulations, we could not administer a hard data collection stop for the participants from the University of Porto, and therefore we preregistered to collect data of *at least* 30 participants from each side.

4 The total number of invited participants at the University of Geneva was matched based on the final number when closing the experiment at the University of Porto. Because of two no-shows in Experiment 1, this resulted in a total of 42 participants from the University of Geneva.

5 Because of an error in communication, no demographic data were collected at the University of Porto. However, the pool of participants consists of mostly young adults with a mean age of about 20 years.

6 One could argue that a stricter cut-off level of 75% would be more appropriate. When using this stricter exclusion criterion, the results did not change: we observed strong evidence against the interaction in Experiment 1 (*N* = 68, BF_10_ = 0.07) and in Experiment 2 (*N* = 52, BF_10_ = 0.09).

7 Because of a programming error, a part of these instructions were mixed up on screen such that participants would need to put their right index finger on the ‘d’ key and their left index finger on the ‘k’ key. Given that participants generally performed quite well on the interruption task (88% and 80% accuracy in Experiment 1 and 2, respectively), we assume that participants might have followed the more natural order and used their left index finger for the ‘d’ key and their right index finger on the ‘k’ key. Nevertheless, if participants followed the instructions as presented on screen, the task should have been even more demanding to execute which aligns with our main goal.

8 In the original study by Souza et al. (2015), participants were divided into two groups to compare between these intervals in Experiment 1. However, the results demonstrated that the observed effect was not mediated by where in the cue sequence an item was refreshed. Therefore, we did not control for this in the current study.

9 The data were collected online, and thus there was no controlled setting to make sure that participants were complying with suppression instructions. However, the absence of articulatory suppression should not impact the results, as previous studies have observed a refreshing frequency effect for arrays of colors—as we use here—without including articulatory suppression (e.g., Loaiza & Souza, 2022; Souza & Oberauer, 2017).

## Code availability

The experimental files and analysis code are available on OSF: https://osf.io/94rvx/overview

## Declarations

### Author note

This work was supported by the Swiss National Science Foundation [Grant No. PCEFP1_181141 to Evie Vergauwe and 100014_192204 to Klaus Oberauer] and the Portugese National Science Foundation [Grant No. CEECINST/00159/2018/CP1511/CT0002 to Alessandra S. Souza]. The body of the work was done while Caro Hautekiet was at the University of Geneva (Experiment 1) and the University of Zurich (Experiment 2). Caro Hautekiet is now at the Vrije Universiteit of Amsterdam. Correspondence should be sent to Caro Hautekiet, Vrije Universiteit Amsterdam, Department of Experimental and Applied Psychology, Vrije Universiteit Amsterdam, Amsterdam, 1081 BT, the Netherlands, c.h.hautekiet@vu.nl. All materials, data, analysis scripts, and supplementary materials can be found on OSF (https://osf.io/94rvx/overview). The design, hypotheses, data exclusion, and analysis plan of each experiment was preregistered on OSF: Experiment 1: https://osf.io/nuvyh/overview Experiment 2: https://osf.io/7hd8e/overview

### Open practices statement

The data, analysis scripts and materials for all experiments are available on OSF (https://osf.io/94rvx/overview). The design, hypothesis, data exclusion, and analysis plan of each experiment was preregistered on OSF prior to conducting the research:

Experiment 1: https://osf.io/nuvyh/overview

Experiment 2: https://osf.io/7hd8e/overview
