# Supplementary material for: Refreshing boosts memory but does not protect it against interference
Source: Psychon Bull Rev. 2026 Feb 17;33(3):60. doi: 10.3758/s13423-026-02856-0 (PMC13661716; doi:10.3758/s13423-026-02856-0)
Supplement: Supplementary file 1 [file 13423_2026_2856_MOESM1_ESM.docx]

**Supplementary Materials**

1. **Practice trials**

Participants first received instructions about the main working memory task and executed 6 practice trials on this. Next, in Experiment 1, participants received instructions about the parity task and executed 16 practice trials on only the parity task with feedback. In Experiment 2, participants received instructions about the color judgment task (including an example of the different colors and their corresponding label) and executed 32 practice trials on only the color judgment task (approximately 4 trials per color). For the last 10 of these practice trials, participants had to get a minimum of 8 correct responses to be allowed to proceed to the main task. This was done to make sure that participants were properly trained on the color judgment task as a warm vs. cold decision could be considered as more subjective than an odd vs. even decision, as used in Experiment 1. Failing this criterion resulted in another practice block of 32 trials following the same procedure (with a max. of 3 rounds in total). After participants reached the maximum of 96 practice trials, they automatically continued with the main task. In both experiments, participants received feedback during the practice trials to make sure they were using the correct response buttons. Contrary to the experimental trials, these practice trials were not fixed to 2500 ms, such that the trial would end after a response was provided (with max. 2500 ms) and the feedback had been presented.

Finally, participants received instructions about how these two tasks were going to be merged. This was followed by 12 practice trials of the experimental trials (of which half with interference) before starting the experimental trials (i.e., three trials with interference and three trials without interference). These practice trials were excluded from the subsequent analyses. On average, each trial included three items that were not cued (0-Refreshed items), two items that were cued once (1-Refreshed items), and one item that was cued twice (2-Refreshed items).

2. **Exploratory Analyses**

For both experiments, we preregistered two exploratory analyses. All exploratory analyses were executed using the BayesFactor package (Morey & Rouder, 2018) in R with its default settings.

First, we ran two Bayesian paired two-sided t-tests to examine whether there is a difference in (1) recall error and (2) vulnerability score between a 2-Refreshed item that was cued in the last refreshing step vs. a 2-Refreshed item that was not cued in the last refreshing step. The results are reported in Table S1. For both experiments, we observed substantial evidence for a difference in recall performance between these two types of 2-refreshed items^[1]^ (see Figure S1). Furthermore, we examined whether there is a difference in the vulnerability score (recall error interruption task present minus recall error interruption task absent) between the 2-refreshed items in the last cue position and the not-last cue position, but both t-tests remained inconclusive (see Table S1).

**Table S1**

*Bayes Factors (BF_10_) of Preregistered Exploratory T-Tests on Averaged Recall Error and Vulnerability Score.*

|  |  | **Experiment 1** | **Experiment 2** |
| --- | --- | --- | --- |
| **T-tests** | Error_2-refreshed, last_ ≠ Error_2-refreshed, not-last_ | 42.50 | 279.48 |
|  | Vuln._2-refreshed, last_ ≠ Vuln._2-refreshed, not-last_ | 1.08 | 0.79 |

*Note*. The Bayes Factors (BFs) reflect evidence in favor of or against the presented assumption. When the BF presents conclusive evidence in favor of the tested effect (BF_10_ > 3), the text is presented in green; when the BF reflects inconclusive evidence for or against the tested effect (BF_01_ < 3 or BF_10_ > 0.33), the text is presented in black. Vuln. = Vulnerability Score.

**Figure S1**

*Average Recall Error (in Degrees) for Experiment 1 and 2, as a Function of the Position of the 2-Refreshed Items and Interruption Task.*

*Note.* Average recall error (in Degrees) for Experiments 1 and 2 with standard error bars and individual scores (grey).

Secondly, we investigated whether an item, presumably residing in the focus of attention at the moment the interruption task is presented (i.e., last-refreshed items) is impacted differently by the interruption task than information that is not assumed to be in the focus of attention at any point during the retention interval (i.e., not-refreshed items) and information that is no longer in the focus of attention at the moment the interruption task is presented but has been at some point during the retention interval (i.e., not-last-refreshed items). To do so, we ran a Bayesian repeated measures ANOVA on recall error with Refreshing Status (3 levels; Last-refreshed, Not-last-refreshed, Not-refreshed) and Interruption task (2 levels; Present, Absent) as within-subjects variables. The results of this analysis are reported in Table S2. For both experiments, the best model consisted of the two main effects, and we observed moderate to strong evidence against the inclusion of the interaction in this best model (see Figure S2).

**Table S2**

*Bayes Factors (BF_10_) of Preregistered Exploratory Bayesian ANOVA on Averaged Recall Error.*

|  |  | **Experiment 1** | **Experiment 2** |
| --- | --- | --- | --- |
| **BANOVA** | Refreshing Status | 1.14 x 10^10^ | 4.07 x 10^11^ |
|  | Interruption Task | 393 | 53.52 |
|  | Interaction | 0.05 | 0.14 |

*Note*. Bayes factors (BFs) resulting from model comparison between het best model and the model excluding the effect/interaction (if it is included in the best model) or including the effect/interaction (if it is not part of the best model). The BFs reflect evidence in favor of or against a main effect or interaction. The ‘Interaction’ row refers to the full model including the interaction. When the BF presents conclusive evidence in favor of the tested effect (BF_10_ > 3), the text is presented in green; when the BF reflects conclusive evidence against the tested effect (BF_10_ < 0.33, which corresponds to BF_01_ > 3), the text is presented in red.

**Figure S2**

*Average Recall Error (in Degrees) for Experiment 1 and 2, as a Function of the Cue Position of the Items and Interruption Task.*

*Note.* Average recall error (in Degrees) for Experiments 1 and 2 with standard error bars and individual scores (grey).

All together, these analyses demonstrate that (1) there is a performance benefit for an item that is refreshed twice with the 2^nd^ refreshing opportunity being the last cue position, compared to an item that is refreshed twice, but for which the second refreshing opportunity was during the penultimate cue position, (2) there is no difference in the vulnerability score of items that were refreshed the second time during the last cue position and items that were refreshed the second time during the penultimate cue position, and (3) an item that is presumably in the focus of attention at the moment the interruption task is presented is not impacted differently by the presence of the interruption task, in comparison to an item that is not assumed to be in the focus of attention at the moment the interruption is presented or an item that is no longer in the focus of attention at that time. Taken together, these findings are consistent with our overall conclusion that an item’s susceptibility to interference is not modulated by refreshing.

1. In Experiment 1, the probe corresponded to the last-refreshed item in 3351 trials and to the not-last-refreshed item in 2081 trials. In Experiment 2, the probe corresponded to the last-refreshed item in 2710 trials and to the not-last-refreshed item in 1616 trials.
